# Isolation of *Mycobacterium avium* and other nontuberculous mycobacteria in chickens and captive birds in peninsular Malaysia

**DOI:** 10.1186/s12917-020-02695-8

**Published:** 2021-01-07

**Authors:** Abdul Sattar, Zunita Zakaria, Jalila Abu, Saleha A. Aziz, Gabriel Rojas-Ponce

**Affiliations:** 1 Faculty of Veterinary and Animal Sciences, Lasbela University of Agriculture, Water and Marine Sciences, Uthal, Balochistan 90150 Pakistan; 2 Department of Pathology and Microbiology, Faculty of Veterinary Medicine, Universiti Putra Malaysia UPM, 43400 Serdang, Selangor Malaysia; 3 Institute of Bioscience, Universiti Putra Malaysia, 43400-UPM, Serdang, Selangor Malaysia; 4 Department of Clinical Studies, Faculty of Veterinary Medicine, Universiti Putra Malaysia UPM, 43400 Serdang, Selangor Malaysia; 5 Department of Electrical and Computer Engineering, University of Alberta, Edmonton, T6G 2V4 Canada

**Keywords:** Avian tuberculosis, *Mycobacterium avium subsp. avium*, Culture, Löwenstein Jensen, Cetylperidinium chloride, PCR, IS*901*, Peninsular Malaysia

## Abstract

**Background:**

*Mycobacterium avium* complex (MAC) causes a chronic infectious in the birds known as avian mycobacteriosis. Almost all species of the birds are susceptible to MAC which consists of two closely related species of mycobacteria, that is, *M. avium* and *M. intracellulare.* This study aimed to determine the occurrence of *Mycobacterium avium subsp. avium* (MAA) in chickens and captive birds in selected states of Peninsular Malaysia.

**Results:**

A 300 fecal samples were collected from village chickens (*n* = 100), layer chickens (*n* = 100) and captive birds (*n* = 100). Fecal samples were split into two aliquots for microbiological and molecular detection of MAA. Microbiology detection consisted of microscopy (Ziehl-Neelsen staining) and culture of samples decontaminated with 1% Cetylperidinium chloride and vancomycin, nalidixic acid and amphotericin B (VNA) antibiotic cocktail [vancomycin (VAN) 100 μg/ml, nalidixic acid (NAL) 100 μg/ml and amphotericin B (AMB) 50 μg/ml] onto Löwenstein-Jensen (L-J). Molecular detection (PCR-IS*901)* was performed to detect MAA DNA from the feces and PCR-16S rRNA and IS*901* for identification of genus *Mycobacterium* and *Mycobacterium avium* sub species *avium* isolated onto L-J. All samples (296) were AFB negative smear. *M. avium* was isolated in 0.3% (1/296) samples by culture and detected in 2.5% (6/242) samples by PCR (IS*901*). Other mycobacteria were found in 1.7% (5/296) chickens. Of five isolates, two were identified as *Mycobacterium terrae* and *M. engbaekii* and remaining isolates were not sequenced. Birds positive for *M. avium* included White Pelican (*n* = 1) Black Hornbill (*n* = 1), Macaw (*n* = 2), Cockatoo (*n* = 2) and village chicken (*n* = 1).

**Conclusion:**

It is concluded that chickens and birds were infected with *M. avium* in selected areas of Peninsular Malaysia. Although, PCR is rapid, reliable and cost effective method for detection of *M. avium* in a subclinical stage, the culture of the avian feces should still be used as a reference test for the diagnosis of avian tuberculosis.

## Background

*Mycobacterium avium* complex (MAC) is an opportunistic pathogen and ubiquitous in the environment that cause tuberculosis in the birds [1, 2]. Avian tuberculosis causes irreparable losses to rare and endangered avian species such as wild birds, captive pet birds and domestic hen [2, 3]. Age, congested population of birds and unhygienic conditions are considered the major predisposing factors [4]. Modern poultry husbandry practices have significantly reduced the incidence of tuberculosis in commercial poultry [2, 3]. However, sporadic outbreaks have been reported in commercial chicken and duck flocks at different times [1, 5].

Poultry meat is the major source of the cheap protein to the multi ethnic population in Malaysia [6]. The total poultry population in Peninsular Malaysia was estimated to be 161.3 m broiler, 56.7 m layer, 17.8 m breeder, 12.8 m local chicken and 9.6 m ducks [7]. The share of indigenous chickens and ducks in the poultry population is estimated as 5.1 and 1.15% respectively [7]. Large proportion of villagers in Peninsular Malaysia is involved in raising indigenous chicken [8]. Despite that village based poultry plays a significant role in improving the nutritional status, income, food security and livelihood of many small holders [9], some infectious diseases such as Avian influenza, Newcastle disease, infectious bursal disease and salmonellosis are the major cause of high mortality and low production of the poultry from different part of the world and Malaysia [10–13]. Although, avian tuberculosis is also another important infectious diseases that has been reported from different poultry in the world [1, 14–16], there is no published data about the prevalence of this infectious disease neither in poultry nor in captive birds from Malaysia.

Diagnosis of avian tuberculosis can be made by histopathology, light microscopy and isolation at postmortem [17] and from live birds by using feces as noninvasive source [18]. Light microscopy is the cheapest and rapid method to detect mycobacteria, but it has very low sensitivity [19]. Isolation of mycobacteria on culture media is the gold standard [19, 20], however, the main drawback of culture is its low sensitivity. Sensitivity of culture depends on the number of organism present in the samples [20]. Liquid media are preferred over conventional egg based solid media such as Löwenstein-Jensen (L-J) due to rapid detection [21]. However, high cost of the liquid media has limited their routine use [19]. L-J culture is cost effective and is frequently used for culture in low resource settings [19]. L-J recovers more positive cultures and more colonies on positive samples than L-J modified with antibiotics and Herald’s egg yolk medium [22]. Additionally, sensitivity of primary culture for mycobacteria can be improved when a gentle chemical agent is used to kill unwanted bacteria present in the specimen [23]. Studies have shown that the Cetylperidinium chloride – Sodium chloride (CPC) method is favorable alternative method to isolate mycobacteria [24, 25] as it is less toxic to mycobacteria [24, 25], more effective in controlling culture contamination [23, 24], supports early growth of mycobacteria [23, 26] and does not need neutralization as its inhibitory effects are neutralized on egg based media [27]. Likewise, the incorporation of a VNA antibiotic cocktail composed by vancomycin (VAN), nalidixic acid (NAL) and amphotericin (AMB), in the decontamination of sample has improved the sensitivity of the primary culture (CPC 27.8% and CPC-VAN 66.7%) and reduced the contamination of cultures (CPC 66.7% % and CPC-VAN 19%) [24]. Polymerase chain reaction (PCR) is reasonable alternative to conventional methods for the detection of slow growing mycobacteria [28]. PCR is rapid, reliable, less laborious, and cost effective [29]. Previous studies have shown that PCR targeting IS*901* gene detects most of the pathogenic mycobacteria [30]. PCR is more sensitive than culture to detect mycobacteria in the samples containing low number of mycobacteria [14]. Kriz and coworkers (14) detected *M. avium* sub species *avium* and *M. avium* from tissue samples of cochin hen using quantitative real time PCR by amplification of genes IS*901* and IS*1245*.

In order to determine the presence of avium tuberculosis in birds, this study reported the occurrence of *M. avium* subsp. *avium* (MAA) in chickens and captive birds from selected states of Peninsular Malaysia by isolating MAA from avian fecal samples on L-J culture and by direct polymerase chain reaction (PCR) by amplifying insertion sequence IS*901* [14, 31].

## Results

### Isolation of mycobacteria

**Fig. 1:**
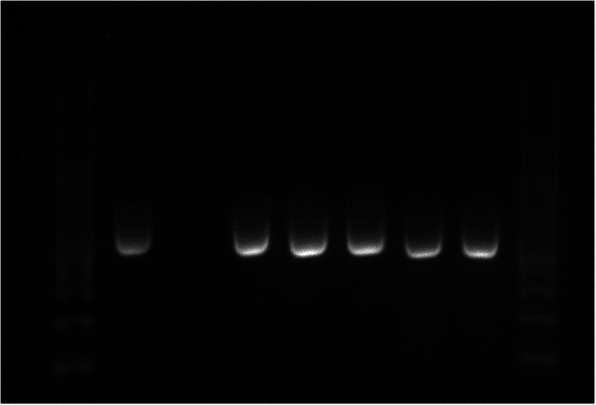
Agarose electrophoresis of PCR (16S rRNA) product of mycobacteria isolated from feces. M, 100 bp size molecular DNA marker; C+ positive control; C- negative control; 1 to 5 (bp-564) isolates

**Fig. 2:**
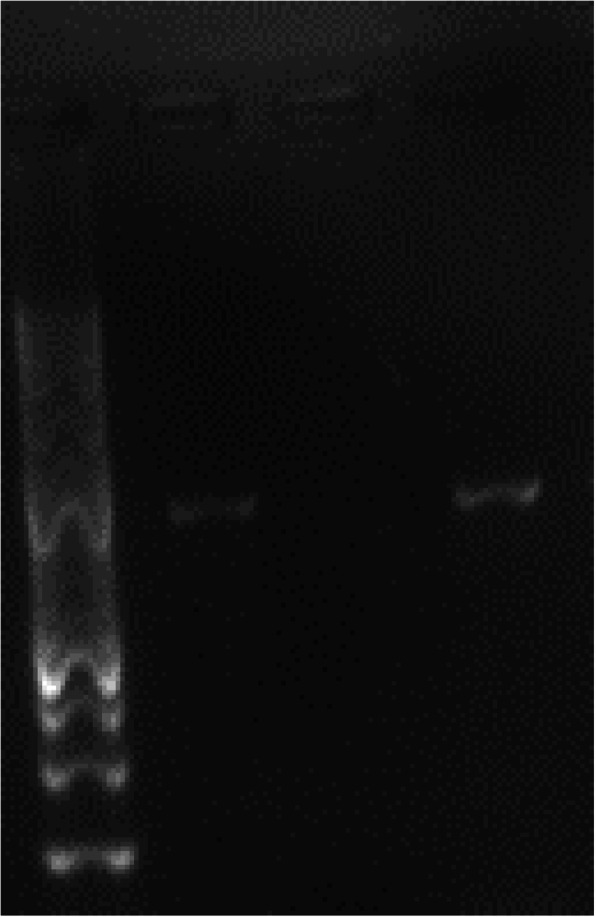
Agarose electrophoresis of PCR (IS*901*) product of *Mycobacterium avium* isolated from *Pelicanus onocratalus*’ feces. Other negative IS*901* samples are not shown on the gel. M, 100 bp size molecular DNA marker; C+ positive control; C- negative control; 1 (bp-753)

Of 300 fecal samples, 296 samples (888 replicates) were cultured and 4 samples were excluded from culture because of the small quantity of feces and were used only for PCR detection (Table 1). Distribution of 296 fecal samples used for culture was as village chickens (*Gallus domesticus*) (100), layer chickens (100) and captive birds (96). Six (6) L-J cultures (6/296, 2%) were AFB-positive. Colonies were seen within two to three weeks of incubation. Of six (6) AFB positive isolates, five (5) were identified as mycobacteria by PCR 16S rRNA (Fig. 1) and remaining one AFB isolate was not amplified due to insufficient colonies. Further amplification of five (5) mycobacterial isolates by PCR IS*901,* only one isolate was amplified as *M. avium* subsp. *avium* (Fig. 2) which was further confirmed by sequencing. This isolate came from a white pelican (*Pelicanus onocrotalus*). Two amplicons of PCR 16S-rRNA were sequenced and identified as *Mycobacterium terrae* and *Mycobacterium engbaekii* (Table 1). Sequencing was not performed for remaining two isolates because both isolates did not encode for IS*901* sequence, meant that both isolates did not belong to MAA. No mycobacteria were isolated from layer chickens (Table 1). The partial sequences can be accessed at NCBI using the accession numbers MH428008 (*Mycobacterium avium*), MH333265 *Mycobacterium terrae* (*Mycolicibacter terrae*) and MH333264 *Mycobacterium engbaekii* (*Mycolicibacter engbaekii*).

**Table 1:** Occurrence of *Mycobacterium avium* in chickens and birds in selected area of Peninsular Malaysia

Farm ID	Total samples (*n*)	Culture on L-J						Direct PCR (IS*901)*		
		◆Samples for culture (*n*)	Replicates (*n*)	Culture positive (%)	Identification of isolates	Contaminated cultures (replicate %)	Species of birds	Samples for PCR (*n*)	Positive (%)	Species of birds
F1*	4	4	12	0		33.3%		4	0	
F2*	29	29	87	S0		0%		8	0	
F3*	12	12	36	0		5.6%		5	0	
F4*	12	12	36	0		100%		11	1 (9.0)	*Gallus domesticus*
F5*	23	23	69	0		14.5%		16	0	
F6*	4	4	12	2 (50)	*M. terrae, M. engbaekii*	66.7%	*Gallus domesticus*	4	0	
F7*	16	16	48	2 (12.5)	Mycobacterium spp. (2 isolates)^a^	45.8%	*Gallus domesticus*	10	0	
F8^#^	11	11	33	0		69.7%		11	0	
F9^#^	11	11	33	0		57.6%		11	0	
F10^#^	35	35	105	0		30.5%		35	0	
F11^#^	43	43	129	0		23.3%		43	0	
F12^҂^	5	5	15	1 (20)	*M. avium*	20.0%	*Pelecanus onocrotalus*	2	0	
F13^҂^	12	12	36	0		11.1%		11	2 (18.2)	*Ara* spp. and *Cacatua* spp.
F14^҂^	15	15	45	0		33.3%		11	2 (20)	*Ara* spp. and *Anthracoceros malayanus*
F15^҂^	21	20	60	0		1.7%		21	1 (4.7)	*Cacatua* spp.
F16^҂^	8	8	24	0		0%		0	0	
F17^҂^	3	2	6	0		33.3%		3	0	
F18^҂^	11	11	33	0		30.3%		11	0	
F19^҂^	25	23	69	1 (4,3)	Mycobacterium spp. (1)^b^	21.7%	*Phigys solitaries*	25	0	
Total	300	296	888	6 (2.0)		248/888 (27.9%)		242	6 (2.5)	

*Village chickens, ^**#**^Layer chickens and ^**҂**^captive exotic birds. No correlation was seen among the positive samples by culture and direct PCR. PCR was specific to *M. avium* (IS*901*). ◆Z-N stain of all concentrated avian fecal samples were negative. ^a^ sequencing was not performed for these isolates. ^b^ this isolate was positive for Z-N staining and PCR was not performed

### Contamination of cultures

Overall proportion of contamination was 27.9% (248/888 replicates) (Table 1). Proportion of culture contamination was 35.3% (106/300), 34.7% (104/300) and 13.1% (38/288) for village chicken (F1 to F7), layer chicken (F8 to F11), and exotic captive birds (F12 to F19), respectively. Contamination of primary cultures appeared as mucoid white or yellow creamy colonies causing blue discoloration and liquefaction of L-J green slants. Contaminating colonies covered the surface of the L-J slant and most of them grown during first week of incubation. Fungal contamination appeared after 4th week of incubation. Identification of contaminating organisms was not performed as it was beyond the scope of this study.

### Detection of *Mycobacterium avium* DNA from fecal samples

**Fig. 3:**
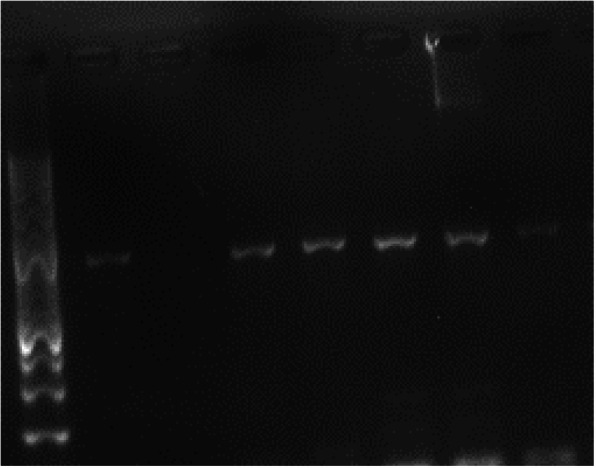
Agarose electrophoresis of PCR (IS*901*) product of *Mycobacterium avium* detected from a batch of five DNA samples extracted from avian feces. M, 100 bp size molecular DNA marker; C+ positive control; C- negative control; 1 to 5 bp-753

**Fig. 4:**
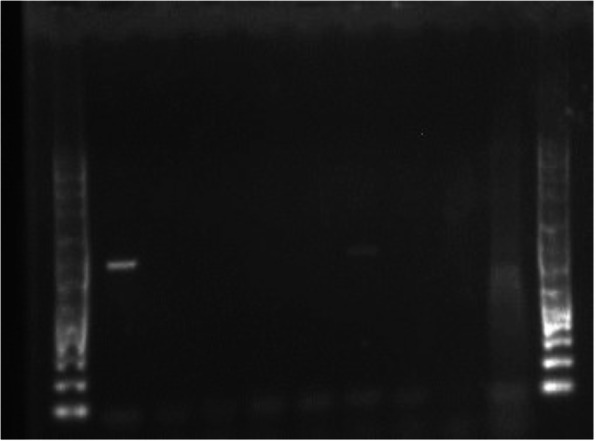
Agarose electrophoresis of PCR (IS*901*) product of *Mycobacterium avium* isolated from a batch of seven DNA samples extracted from avian feces. M, 100 bp size molecular DNA marker; C+ positive control; C- negative control; line 4 (4th sample) was positive from a Black hornbill (*Anthracoceros malayanus*) and other fecal samples were negative

**Fig. 5:**
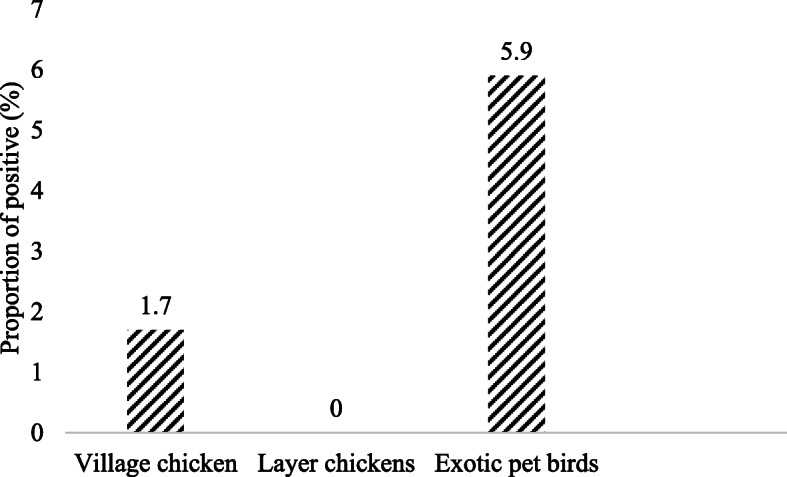
*Mycobacterium avium subsp. avium* detected by PCR IS*901* from avian fecal samples

Two hundred and forty two (242) out of 300 fecal samples were processed by PCR (Table 1). Remaining 58 samples were excluded from PCR detection because 20 fecal samples were only enough for culture and DNA extraction from 38 samples was not successful (no visible DNA yield in the agarose electrophoresis) perhaps due to technical issues like poor lysis ability of QIAamp® Fast DNA Stool Mini Kit (Qiagen®) and 5 min incubation of feces InhibitEX homogenate on heating block. Distribution of the samples was; village chicken (*n* = 58), layer chicken (*n* = 100) and exotic captive birds (*n* = 84) (Table 1). PCR (IS*901*) detected 753 bp fragment of IS*901* of *M. avium* in 2.5% (6/242) birds (Figs. 3 & 4). Proportion of the positive exotic captive birds and village chickens were 5.9% (5/84) and 1.7% (1/58) respectively (Fig. 5). No *M. avium* was detected in the layer chickens (Fig. 5). PCR positive birds included Macaw *Ara* spp. (*n* = 2), Cockatoo *Cacatua* spp. (*n* = 2), Black Hornbill *Anthracoceros malayanus* (*n* = 1) and village chicken *Gallus domesticus* (*n* = 1) (Table 1).

### Risk factor analysis

Risk factors analysis was performed for those flock size which showed positive samples. Univariate analysis of the risk factors revealed that flock size < 50 versus > 500 (OR = 11.47 *P* = 0.01 Cl: 1.31–100.3) and breed of the birds (local chickens verses exotic captive birds) (OR = 0.103; *P* = 0.02; Cl: 0.012–0.89) were significant predictors of *M. avium* occurrence in the birds (Table 2). Although feed [32], water [31] and free birds [33] are considered main reservoirs of MAA, in this study, feed (*P* = 0.07), source of water (*P* = 0.09) and access of free birds were not statistically significant risk factors.

**Table 2:** Analysis of risk factors using PCR as dependent variable

Factor	Categories	Prevalence (%)	*P*-value	OR	95%Cl
Location	F4*	1/11 (9)		1.0	Ref
	F13^#^	2/11 (18.2)	0.5	0.450	0.035–5.843
	F14^#^	2/12 (20)	0.5	0.5	0.039–6.43 s
	F15^#^	1/21 (4.7)	0.5	2.0	0.113–35.41
No of birds	< 50	5/67		1.0	Ref
	> 500	1/141	0.01^a^	11.47	1.313–100.3
Type of the birds	Village chicken	1/57		1.0	Ref
	Layer chicken	0/100	–	–	NA
	Captive pet birds	5/85	0.22	.28	0.03–2.512
Breed of birds	Local chicken	1/157			Ref
	Exotic captive birds	5/85	0.02^a^	0.103	0.012–0.893
Feed	Commercial	2/168			Ref
	Local	4/74	0.07	0.21	0.038–1.178
Source of water	Open surface tank	4/80			Ref
	Drinkers	2/162	0.09	4.21	0.75–23.495
Hygiene	Good	3/131			Ref
	Fair	3/71	0.35	0.531	0.104–2.704
	Poor	0/40			
Access of wild birds	Yes	5/122			Ref
	No	1/120	0.1	5.08	0.585–44.191

* Village chicken; ^#^ exotic captive birds; ^a^ significant difference; *Ref* reference category, *NA* not applicable, *OR* Odd ratio, *Cl* Confidence interval. Facilities F1–3, F5–12 and F16–19 were not included in the analysis as there was zero prevalence in the facilities

### Sequence analysis

BLAST analysis of two sequences of 16S rRNA amplicons revealed identity index 100% with *M. terrae* and *M. engbaekii*. All DNA sequences of IS*901* showed identity index ranging from 98 to 100% and 89 to 94% query coverage with *M. avium* isolates in NCBI GenBank.

### Phylogenetic analysis

**Fig. 6:**
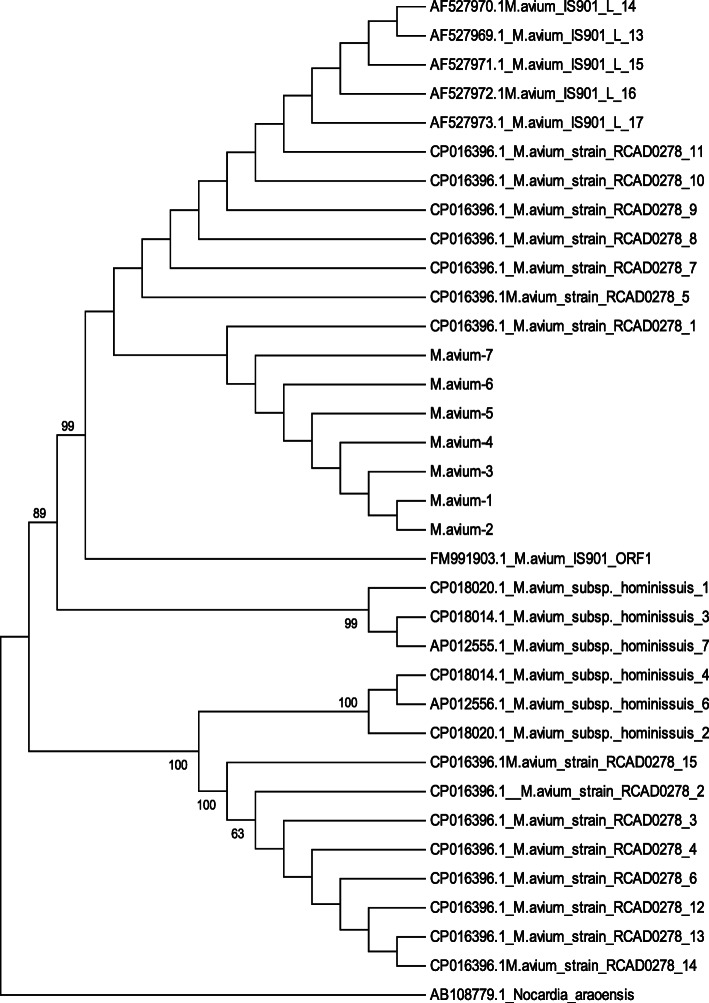
A maximum likelihood phylogentic tree showing the relation between strains of *Mycobacterium avium* (IS*901* gene). Phylogenetic analysis was performed using Mega6. The analysis was carried out based on IS*901* nucleotide sequences. Analysis showed that DNA sequences of *M. avium* strains obtained in this study were closely related to each other and to *M. avium* strain RCAD0278. The starins of *M. avium* RCAD0278, *M. avium* cervine strain JD/88, *M. avium* ORF1 and *M. avium huminsiuss* from human were grouped in separate clusters. ^a^ Significant evolutionary divergence within the group, ^b^ significant evolutionary divergence between *M. avium* isolated in this study and isolates of *M. avium* retrieved from Genbank. ^c^ Evolutionary divergence within and between the groups was not significant

Phylogenetic analysis revealed that all *M. avium* isolates separated into three main groups and each group was further divided into sub groups. The sub groups were (1) RCAD 0278 and JD88/118 strains, (2) isolates of this study, (3) FM991903 ORF, (4) *M. avium huminissius*, (5) *M. avium huminissius*, and (6) RCAD 0278 strain. DNA sequences of the current study were grouped in one sub group with *M. avium* strain RCAD 0278_1. Reconstruction of the Phylogenetic tree was supported with bootstrap values ranging from 63 to 100% (Fig. 6). Average evolutionary divergence over sequence pairs within the groups revealed that the number of base substitutions per site from averaging over all sequence pairs within sequences of the current study (*P* = 0.01) and isolates of *M. avium* JD 88/188 (*P* = 0.001) were significant. On the other hand, estimates of evolutionary divergence over sequence pairs between groups showed that the number of base substitutions per site from averaging over all sequence pairs between isolates of the current study and isolates of *M. avium* JD88/188 (P = 0.01) and *M. avium* ORF1 (*P* = 0.003) was significant. Evolutionary divergence between the sequence pairs of the isolates of the current study and *M. avium* RCAD02781 (*P* = 0.5) and isolates of *M. avium huminissius* (*P* = 0.4) were not significant.

## Discussion

The results of this study showed that MAA 0.03% (1/296) and 2.5% (6/242) were detected by culture and PCR, respectively. Other environmental mycobacteria 1.7% (5/296) detected by culture were endemic in the birds from Selangor, Melaka, Kuala Lumpur and Putrajaya in Peninsular Malaysia. This study showed that water (*P* = 0.07) and food (*P* = 0.09) were not significant risk factors for *M. avium* and other mycobacteria infection in these birds. However, previous studies have shown that water from different sources (rivers, dams, ponds, lakes, kitchen sediments and bath rooms) is the main reservoir for nontuberculous mycobacteria [31]. In this study, majority of the birds infected with MAA comprised of captive birds as they are more susceptible to MAA mainly due to their relatively long lifespan which increases the accumulated risk of exposure and long incubation period of the pathogen [2, 3]. Although, access of wild birds to the premises of the chickens and birds was not significant risk factor (*P* = 0.1), previous studies have shown that wild birds play important role in the dispersion of pathogens including MAA [34, 35]. Furthermore, the risk analysis may be underestimated due to sample size, low incidence rate and low sensitivity of the current testing methodologies to detect mycobacteria from infected birds.

The laboratory tests used in this study showed some agreement with previous reported studies [22, 36]. Z-N test was not optimal to detect all MAA by culture and PCR. Although the smears were prepared from concentrated pellet sediments, it did not improve the sensitivity of microscopy results. Since that the sensitivity of microscopy is around 10^4^ CFU/ml [35], the negative results for microscopy allow us to estimate that the concentration of AFB in the avian fecal samples could be less than 10^4^ CFU/g of feces. Results of the current study are in line with other studies that reported a low sensitivity of microscopy from duodenal aspirates (5.8%) [37] and fecal samples from Japanese quails (7.2%) [22].

Culture method could detect 2.0% (6/296) of mycobacteria identified as *M. avium*, *M. terrae*, *M. engbaekii* and three other isolates as *Mycobacterium* spp. Although the recovery of mycobacteria on L-J culture was very low, we postulate that this low isolation rate may reflect the real occurrence of mycobacteria in chickens and birds. Furthermore, CPC-VNA decontamination method may has a favorable impact on culture sensitivity by reducing the contamination rate [14, 23–25, 36] and increasing the number of positive AFB culture. Isolation of environmental mycobacteria like *M. avium*, *M. terrae* and *M. engbaekii*, which do not possess IS*901,* may not be pathogenic to the birds because no clinical relevance of these species have been reported [38]. However, isolation of such species of mycobacteria in this study shows that CPC-VNA method allows the isolation of other mycobacteria. Regarding the contamination rate, this study reported that the sample collection procedure and immediate process of the samples had effect on the contamination rate. Fecal cultures from captive birds, in which feces were collected with minimal contamination showed less contamination than cultures from village chicken and layer chicken feces. High proportion of contaminated cultures from the village chickens and layer chickens shows the implications of sample collection procedure as well as immediate process of the samples. Village chickens were free in the fenced area or around the house buildings, therefore sample collection procedure with minimal contamination adopted for this study was not applicable to village chickens. Majority of the samples from village chickens were collected after feces were deposited on ground. Hence, contamination rate was high in the cultures from village chicken. Samples from layer chickens were collected aseptically as they were kept in cages. However, samples that were not processed on the same day of sample collection due to logistic issues (e.g., transportation). Samples from layer chickens were stored at − 20 °C for 1 week before being processed. Previous studies have shown that time for samples processing is important factor affecting primary isolation of mycobacteria [18]. Given that our previous study [24] reported that CPC-VNA can control the contamination rate of L-J cultures better than other methods, we postulate that the contamination rate (27.9%) of L-J cultures in this study is acceptable.

Regarding the use of PCR IS*901* as a rapid method to detect MAA than using culture [14, 38], this study reported that 2.5% (6/242) of MAA DNA from fecal samples was detected by PCR IS*901*. Likewise, other authors such as Kriz et al. [14] detected MAA from intestine and fecal samples of domestic fowl and other birds only by PCR, but not culture. In this study, the rapid detection of MAA by PCR IS*90* is an advantage in order to taking actions of prevention of dissemination and transmission of mycobacteria [39]. Likewise, the current study showed that sequencing of amplified fragment is an affordable potential tool for species identification of mycobacteria [40].

Differences in the detection of MAA by using culture, and further speciation by PCR (16S rRNA), against direct detection of MAA using PCR (IS*901*), can be explained by the difference between sensitivity of the culture and PCR. High sensitivity of PCR can be explained by its ability to detect DNA from viable as well as from dead organism [32] unlike culture that only detect viable organisms. In that way, the toxic effect of CPC-VNA method is a variable that has a negative effect on the sensitivity of culture to detect viable organisms [25, 27]. Other authors have also reported differences in the detection of MAC by culture and PCR. Kriz and coworkers [14] detected *M. avium* subsp*. avium* in tissue samples of 80% Cochin hen, in intestine and fecal samples of 60% of hens and, 20% of the raptors (feces) by PCR using IS*901* gene unlike culture samples that were negative. In another study, Klanicova et al. [30] detected three sub species of *M. avium* in raw and processed meat by PCR using IS*901* and IS*1245* genes. However, cultures of these meat samples were negative for mycobacteria. Douarre et al. [32] also reported that PCR IS*900* detected (36.2%) *M. avium* subsp*. paratuberculosis* in bovine feces, while only 7.9% PCR positive samples were culture positive. Keeping in view the findings of the current study as well as previous studies [14, 30, 32], PCR can be used as a rapid alternate to culture for diagnosis of slow growing mycobacteria [41]; however, this test cannot exclude LJ culture as it is considered gold standard to detect viable organisms (19).

Another interestingly finding reported in this study is the detection of MAA from the fecal sample of White Pelican (*Pelecanus onocrotalus)* sample using culture and Black Hornbill (*Anthracoceros malayanus)* using direct PCR. Although MAA has been reported in domestic hen [42], macaw [16, 43], cockatoo [16, 43], and commercial ducks [1] from different region of the world, to the best of our knowledge only one study [43] reported MAA from White Pelican but there is no published data from Black Hornbill.

Among some limitations of the study was that the infection of the birds with *M. avium* and other species of mycobacteria should be confirmed by postmortem and histopathology [17], however, in the current study, postmortem and histopathology could not be performed. Another limitation was the quantity of feces and procedure for collection of fecal samples. One gram of feces was required for culture and 200 mg for DNA extraction. However, the required quantity of feces from all village chicken (free range) and exotic captive birds could not be collected. Village chickens were free, deposited feces on ground which were exposed to environment, therefore a larger portion of feces discarded. On the other hand, feces of the small pet birds is normally small in quantity. Other limitations that had effect on the sample size were the limited availability of chickens older than one year and exotic captive birds so a total of 300 birds were sampled rather than the calculated sample size of 382. Convenient sampling methods were adopted for selection of farms (bird facilities) due to logistic difficulties. Therefore, occurrence of MAA may be underestimated due to sample type and small sample size. Nevertheless, this preliminary study with limited resources with small sample size reports the occurrence of *M. avium* and other environmental mycobacteria in chicken and captive pet birds in Peninsular Malaysia. Moreover, since Peninsular Malaysia is large country and chicken and captive birds were conveniently sampled from small number of locations which may not be representative of the whole country. Future studies must include a large study area with larger sample size so as the result can be extrapolated to whole population. Furthermore, another limitation of the current study is that causal effect of the identified risk factors cannot be estimated because the cross sectional design of the study which does not allow to determine the temporal sequence of the cause and effect of the risk factors (33).

## Conclusion

This study reports the occurrence of MAA in chickens and birds in the selected areas of Peninsular Malaysia and encourages further studies that include a higher number of birds from same and other locations in Malaysia. The culture of avian feces decontaminated by CPC-VNA method and the direct detection of MAA DNA from fecal samples by PCR IS*901* should be considered as referential tests for the detection of MAA and other members of the genus *Mycobacterium* in further studies.

## Methods

### Reagents

Preparation of CPC, VNA antibiotic cocktail, and L-J culture were described previously [24]. Briefly, CPC (1%) solution was prepared by adding 5 g of CPC (Merck, Germany) and 10 g of NaCl (Merck, Germany) in 500 mL distilled water. VNA working solution consisted of VAN (100 μg/ml), NAL (100 μg/ml) and AMB (50 μg/ml). L-J culture was prepared according to the manufacturer’s instruction (HiMedia, India).

### Sample size

Expected prevalence of avian mycobacteriosis was taken as 50% [35] as there is no previous study on the prevalence of disease in the birds in Peninsular Malaysia. Following formula was used to calculate the required sample size (*N*) [44].

$$ \mathrm{N}=\frac{1.96^2{P}_{exp}\left(1-{P}_{exp}\right)}{d^2} $$

where *N* = required sample size. Pexp = expected prevalence, d = desired absolute precision.

Using the above formula, the estimated sample size was 384 fecal samples. However, due to limitations in sample collection, the sample size was reduced to 300 which included village chicken *Gallus domesticus* (*n* = 100), layer chicken (*n* = 100) and captive birds (*n* = 100). Exotic captive birds included macaw *Ara* spp. (*n* = 13), cockatoo *Cacatua* spp. (*n* = 14), African grey parrot *Psittacus erithacus* (*n* = 23), amazon parrot *Amazona* spp. (*n* = 8), Parakeet *Melopsittacus undulates* (*n* = 4), Blue fronted parakeet *Thectocercus acuticaudatus* (*n* = 1), Lori keets *Phigys solitaries* (*n* = 7), Parakeet rosella *Platycercus* (*n* = 1), pigeon *Columbia domestica* (*n* = 10), Cocktiel *Nymphicus hollandicus* (*n* = 2), Indian ring neck parrot *Psittacula krameri* (*n* = 1), Sunconure *Aratinga solstitialis* (*n* = 2), Green cheek parrot *Pyrrhura molinae* (*n* = 1), Monk parakeet *Myiopsitta monachus* (*n* = 1), White pelican *Pelecanus onocrotalus* (*n* = 5), Curassow *Mitu mitu* (*n* = 1), Black Hornbill *Anthracoceros malayanus* (*n* = 2), Pea hen *Pavo cristatus* (*n* = 1), White Bellied Sea Eagle *Haliaeetus leucogaster* (*n* = 2) and Buffy fish owl *Ketupa ketupu* (*n* = 1). Convenient sampling procedure was adopted for selection of poultry farms as well as the birds. Location of the farm, type and number of birds are summarized in Table 3.

**Table 3:** Location, number and description of chickens and birds

Farm ID	Location	Description	Type of Birds	No of samples
F1	Selangor	Village chicken	Village chicken	4
F2	Selangor	Backyard chicken	Village chicken	29
F3	Selangor	Chicken farm	Village chicken	12
F4	Selangor	Chicken farm	Village chicken	12
F5	Selangor	Chicken farm	Village chicken	23
F6	Selangor	Chicken farm	Village chicken	4
F7	Selangor	Chicken farm	Village chicken	16
F8	Melaka	Layer farm	Layer chicken	11
F9	Melaka	Layer farm	Layer chicken	11
F10	Melaka	Layer farm	Layer chicken	35
F11	Melaka	Layer farm	Layer chicken	43
F12	Putrajaya	Wetland park	Exotic birds	5
F13	Selangor	Private Zoo	Exotic birds	12
F14	Kuala Lumpur	Zoo Negara	Exotic birds	15
F15	Klang	Pet shop	Exotic birds	21
F16	Selangor	Indoor birds	Exotic Birds	8
F17	Putrajaya	Private owner	Exotic Birds	3
F18	Selangor	Private owner	Exotic Birds	11
F19	Selangor	Private owner	Exotic Birds	25
**Total**				**300**

### Study area

**Fig. 7:**
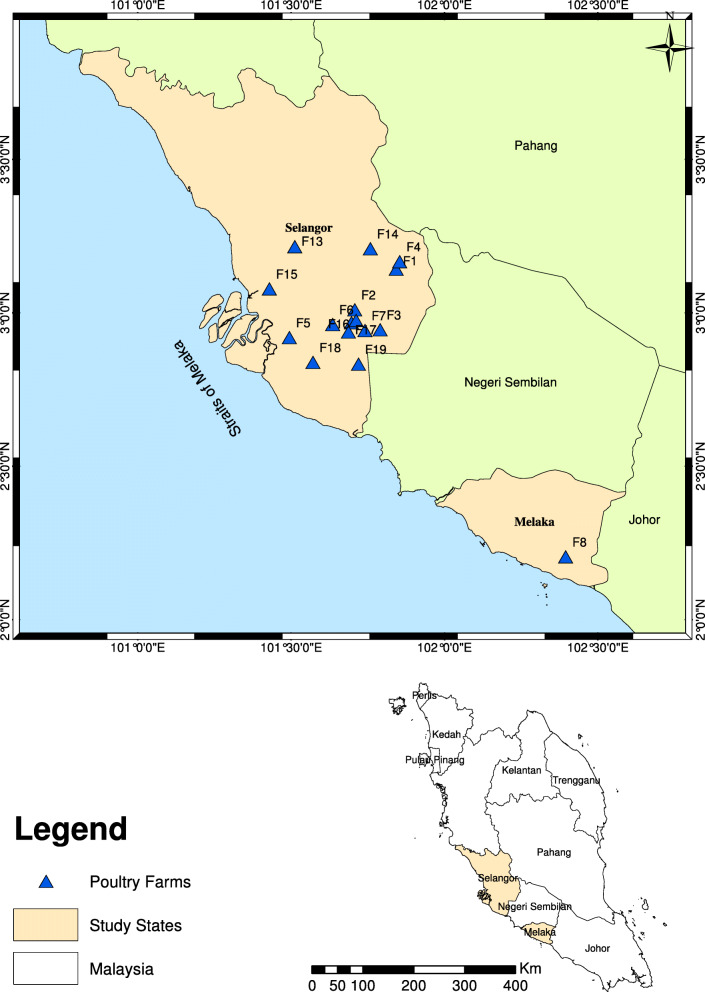
Map of the study area. This map is created using ArcMap version 10.4.1 by putting the coordinates of the sampling sites of the study areas

The study was conducted in the central region of Peninsular Malaysia (Selangor and Melaka states and Federal Territories of Kuala Lumpur and Putrajaya) (Table 3 & Fig. 7). Kuala Lumpur is the national capital and Putrajaya is the federal administrative center of Malaysia. Selangor state is located on the west coast of Peninsular Malaysia. While Melaka state is located in the southern region [45].

### Sample collection

Fecal samples were collected from May 2016 to September 2017. Fecal samples were collected during morning hours. For sample collection with minimal contamination, clean newspapers were spread beneath the cages. Feces were collected in sterilized Falcon tube 50 mL (TPP®, Switzerland) using sterilized metal spatula. An insulated container with ice packs was used to keep the samples cool during transportation to the laboratory. Samples were aliquoted into two, one for microbiology and other for direct PCR detection. However, very small samples were either used for culture or PCR. Sample aliquots for PCR were stored at − 20 °C and samples for microbiology were processed on the same day otherwise stored at 4 °C for not more than 24 h. However, fecal samples from layers were collected by veterinarian at layer farm and were stored at − 20 °C for one week and transported to the laboratory. Farms were categorized into four groups according to the number of birds, the categories were as < 50, 50 to 100, 100 to 200 and > 500.

### Isolation of mycobacteria

#### Preparation of fecal samples

One (1) g of fecal sample was homogenized in 30 mL sterile distilled water in a 50 mL Falcon tube. However, some of the samples which were too small in quantity, less than one gram feces was used. Sample homogenization was performed with vigorous shaking followed by vortex at high speed for 1 min. Then the samples were allowed to stand undisturbed at room temperature for 30 min. Supernatants were filtered through sterile surgical gauze (mesh size 19 × 15 Promedictech Sdn Bhd, Malaysia). Filtrates were centrifuged at 3000×g (Eppendorf AG-22331 Hamburg, Germany) at 10 °C for 20 min. Supernatants were poured off and pellets were resuspended in 1 mL sterile distilled water and mixed well by short vortex. A 100 μL of pellet sediment was used for AFB smear and remaining pellet sediment was subjected to decontamination with CPC.

#### AFB smear preparation

A 100 μL of the concentrated fecal sample (pellet sediment) was placed and spread onto a clean glass slide, allowed to air dry, heat fixed and stained with Ziehl Neelsen (Z-N) stain. Smears were examined under 100X oil immersion magnification.

#### Decontamination

Remained pellet sediment was mixed with 25 mL of CPC (1%) by vigorous shaking and vortex. Then the suspension was allowed to stand at 37 °C for 24 h. The suspension was centrifuged at 3000×g for 15 min at 10 °C and supernatants were decanted and pellets were dissolved in 1 mL of sterile distilled water before mixing with equal volume of VNA working solution and incubated at 37 °C for 24 h. Sediments were mixed by vigorous shaking before inoculating in triplicate onto L-J slants with 100 μL of pellet sediment. Each replicate was considered as an individual culture. L-J slants were placed in a slanted position and incubated at 37 °C for no more than 8 weeks. Mycobacterial colonies were confirmed by Z-N staining followed by PCR amplification of 16S rRNA and IS*901* genes.

### Identification of mycobacteria

#### DNA extraction from isolates

DNA extraction was performed using Blood and tissue kit (Qiagen®) as described previously [24] . Briefly, fresh colonies were lysed in 200 μL lysis buffer incubated at 37 °C for 30 min (heat block-Labnet, USA). To digest the contaminating proteins and lyse all cells, 20 μL proteinase K and 200 μL buffer AL were added to the mixture and incubated for 10 min at 70 °C which was followed by precipitation of nucleic acid using 200 μL of ethanol 96% (Fisher Scientific, UK). After applying all lysate of each sample to a DNeasy Mini column and centrifugation for 1 min at 5900×g (Profuge 14D, USA), the DNA was washed with 500 μL of buffers AW1 and AW2. DNA was eluted with 50 μL Buffer AE. The quality of DNA was assessed by gel electrophoresis using 0.8% agarose gel. Purified DNA was stored at − 20 °C.

#### PCR amplification

Top *Taq*™ Master Mix (Qiagen®) was used to perform amplification of purified DNA following the manufacturer’s instruction. Briefly, a 25 μL reaction mixture was prepared by adding 12.5 μL Top *Taq* Master Mix 2x, 6.5 μL RNase free water, 5 μL template DNA and 0.5 μL (0.2 μM) of each primer. A 564 bp fragment of 16S rRNA was amplified by 16MycF 5′-CGT GCT TAA CAC ATG CAA GTC G-3′ and 16MycR 5′- GTG AGA TTT CAC GAA CAA CGC-3 [24]. Initially DNA was denatured for 2 min at 95 °C followed by 35 cycles of denaturation at 94 °C for 30 s, annealing at 52 °C for 30 s and extension at 72 °C for 1 min. Final extension was performed for 10 min at 72 °C [24]. Eppendorf Mastercycler (Germany) was used for amplification of DNA. Primer set; IS*901*-F 5′-GAA CGC TGC TCT AAG GAC CTG TTG G-3′ and IS901-R 5′- GGA AGG GTG ATT ATC TGG CCT GC-3′ was used to amplify a 753 bp fragment of IS*901* to identify *M. avium* [24]. Reaction mixture and concentrations were similar as described for 16S rRNA. Amplification conditions included initial denaturation for 3 min at 95 °C and 35 cycles of denaturation for 1 min at 95 °C, annealing for 40 s at 60 °C, extension for 35 s at 72 °C. Final extension was carried out for 10 min at 72 °C [24]. PCR amplification was performed in thermocycler (BIO RAD, USA). *Mycobacterium avium* sub species *avium* (ATCC 15769) and RNase free water (Qiagen®) were included as positive and negative controls in each run of PCR assays.

### Detection of *M. avium* DNA in the feces

#### DNA extraction

DNA was extracted from fresh or frozen fecal samples using QIAamp® Fast DNA Stool Mini Kit (Qiagen®) according to the manufacturer’s instructions. Briefly, 220 mg of fecal sample was homogenized in 1 mL InhibitEX Buffer, incubated for 5 min at 90 °C on a heating block and centrifuged for 1 min at 15600×g. A 600 μL of the supernatant was transferred to a new tube containing 25 μL of proteinase K and then 600 μL of buffer AL was added, mixed by short vortex and incubated for 10 min at 70 °C. After adding 600 μL ethanol (96%) to the lysate and mixing by short vortex, 600 μL of lysate was transferred to QIAamp spin column and centrifuged for 1 min at 15600×g. All lysate was loaded to QIAamp spin column gradually by repeated transfer of lysate and centrifugation. The extract was washed twice with washing buffers AW1 and AW2 and finally DNA was eluded with 50 μL Buffer ATE as described earlier.

#### PCR amplification

*Mycobacterium avium subsp. avium* was detected in feces extract DNA by amplifying a 753 bp segment of IS*901*. Primers, reaction mixture, reagents concentration and thermal protocol were same as described earlier.

#### Gel electrophoresis

PCR products were electrophoresed in 2% agarose (Fischer Scientific, USA) in 0.5X TBE (Sigma-Alorich, Germany) stained with SYBER safe DNA gel stain (Invitrogen, USA). Gel electrophoresis was performed at 80 V for 90 min. AlphaImager™ (alpha Innotech) was used to visualize the gels [24].

#### Sequencing and sequence analysis

PCR amplicons of 16S rRNA and IS*901* were sent to First BASE Laboratories (Malaysia) for sequencing using the same primers as used for DNA amplification. Sequence data were compared by BLAST tool P analysis with bacterial sequences publically available on NCBI GenBank. Percentage of similarity with the reference sequences and query coverage were recorded. Multiple Sequence Alignment was performed using BioEdit version 7.2.5. Mega software (Mega 6) was used to reconstruct the phylogenetic tree using Maximum Likelihood. A *Nocardia asteroids* sequence was used as the out group.

### Statistical analysis

Data was entered in Microsoft excel V.13 and SPSS V.22 was used for data analysis. Descriptive Data was present by frequency tables and percentages. Fischer exact test was used for risk factor analysis and *p* value < 0.5 was considered significant.

## Data Availability

The dataset generated and/ analyzed during the current study are available in NCBI GenBank using the accession numbers MH428008, MH333265 and MH333264.

## Abbreviations

CPC: Cetylperidinium chloride – Sodium chloride
NaCl: Sodium chloride
VAN: Vancomycin
NAL: Nalidixic acid
AMB: Amphotericin B
VNA: Mixture of vancomycin, nalidixic acid and amphotericin B
L-J: Löwenstein-Jensen
Z-N: Ziehl Neelsen
AFB: Acid fast bacilli
MAA: *Mycobacterium avium subsp. avium*
IS*901*: Insertion sequence 901
NCBI: National Centre for Biotechnology Information
BLAST: Basic local alignment search tool
CI: Confidence interval
OR: Odds Ratio
